# Epicardial Adipose Tissue-Derived IL-1β Triggers Postoperative Atrial Fibrillation

**DOI:** 10.3389/fcell.2022.893729

**Published:** 2022-05-05

**Authors:** Serena Cabaro, Maddalena Conte, Donato Moschetta, Laura Petraglia, Vincenza Valerio, Serena Romano, Michele Francesco Di Tolla, Pasquale Campana, Giuseppe Comentale, Emanuele Pilato, Vittoria D’Esposito, Annabella Di Mauro, Monica Cantile, Paolo Poggio, Valentina Parisi, Dario Leosco, Pietro Formisano

**Affiliations:** ^1^ Department of Translational Medical Sciences, University of Naples Federico II, Naples, Italy; ^2^ URT Genomic of Diabetes, Institute of Experimental Endocrinology and Oncology, National Research Council, Naples, Italy; ^3^ Casa di Cura San Michele, Maddaloni, Italy; ^4^ Centro Cardiologico Monzino IRCCS, Milan, Italy; ^5^ Department of Pharmacological and Biomolecular Sciences, University of Milan, Milan, Italy; ^6^ Department of Advanced Biomedical Sciences, University of Naples Federico II, Naples, Italy; ^7^ Pathology Unit, INT-IRCCS Fondazione Pascale, Naples, Italy

**Keywords:** epicardial adipose tissue, cytokines, inflammation, atrial fibrillation, fibrosis, cardiac remodeling

## Abstract

**Background and aims:** Post-operative atrial fibrillation (POAF), defined as new-onset AF in the immediate period after surgery, is associated with poor adverse cardiovascular events and a higher risk of permanent AF. Mechanisms leading to POAF are not completely understood and epicardial adipose tissue (EAT) inflammation could be a potent trigger. Here, we aim at exploring the link between EAT-secreted interleukin (IL)-1β, atrial remodeling, and POAF in a population of coronary artery disease (CAD) patients.

**Methods:** We collected EAT and atrial biopsies from 40 CAD patients undergoing cardiac surgery. Serum samples and EAT-conditioned media were screened for IL-1β and IL-1ra. Atrial fibrosis was evaluated at histology. The potential role of NLRP3 inflammasome activation in promoting fibrosis was explored *in vitro* by exposing human atrial fibroblasts to IL-1β and IL-18.

**Results:** 40% of patients developed POAF. Patients with and without POAF were homogeneous for clinical and echocardiographic parameters, including left atrial volume and EAT thickness. POAF was not associated with atrial fibrosis at histology. No significant difference was observed in serum IL-1β and IL-1ra levels between POAF and no-POAF patients. EAT-mediated IL-1β secretion and expression were significantly higher in the POAF group compared to the no-POAF group. The *in vitro* study showed that both IL-1β and IL-18 increase fibroblasts’ proliferation and collagen production. Moreover, the stimulated cells perpetuated inflammation and fibrosis by producing IL-1β and transforming growth factor (TGF)-β.

**Conclusion:** EAT could exert a relevant role both in POAF occurrence and in atrial fibrotic remodeling.

## Introduction

Atrial fibrillation (AF), the most common cardiac arrhythmia, is the result of electrical and structural remodeling of the atria encompassing interactions among cellular and neurohormonal mediators (Hu et al., 2015). Post-operative AF (POAF), defined as new-onset AF in the immediate period after surgery, is associated with hemodynamic instability, increased risk of stroke, an eightfold increase in the risk of subsequent AF (Ahlsson et al., 2010), and cardiovascular death (Ahlsson et al., 2010; Lee et al., 2014; Melduni et al., 2015).

Mechanisms leading to POAF are not completely understood, but it is probably the consequence of both pre-existing factors, related to atrial remodeling, and peri-operative triggers that induce AF when a vulnerable substrate is present. Inflammation could be one of the most potent AF triggers and the NACHT, LRR, and PYD domain-containing protein 3 (NLRP3) inflammasome myocardial activation, through the production of the pro-inflammatory cytokines interleukin (IL)-1β and IL-18, is associated with the pathogenesis of AF by promoting atrial structural and electrical remodeling (Yao et al., 2018). IL-1β increased gene expression levels are associated with atrial remodeling and sustained AF (Matsushita et al., 2019). IL-1β plays an important role in the inflammatory cascade and coordinates the cellular response to tissue injury, promoting the recruitment of inflammatory cells and the increased production of other cytokines (Dinarello, 2011). Animal studies suggest that IL-1β contributes to myocardial electrophysiological remodeling and its inhibition reduces arrhythmogenesis (De Jesus et al., 2017). Epicardial adipose tissue (EAT), the visceral fat depot of the heart, has been proposed as a local source of inflammatory mediators with a potential role in AF (Arai et al., 2011; Wong et al., 2017). Potential arrhythmogenic mechanisms of EAT in AF include structural remodeling of the atria and arrhythmic trigger by infiltrations of adipose tissue, fibrosis modulation, myocardial inflammation, and oxidative stress (Wong et al., 2017). IL-1β stimulates activin A expression (Arai et al., 2011) that has been reported to be produced by epicardial adipose tissue (EAT) of patients evolving POAF after cardiac surgery (de Kretser et al., 2012; Wang et al., 2019). We recently reported an increased inflammatory status of EAT also in POAF (Petraglia et al., 2022). However, further evidence is required to confirm all these hypothetic mechanisms in order to clarify the role of EAT in cardiovascular disease and AF.

In the present manuscript, we aim at exploring the link between EAT-secreted IL-1β, atrial remodeling, and POAF in a population of coronary artery disease (CAD) patients undergoing cardiac surgery.

## Materials and Methods

### Patient Enrollment and Tissue Collection

Forty patients with coronary artery disease (CAD) undergoing elective coronary artery bypass grafting (CABG) were enrolled at the cardiac surgery unit of the University of Naples “Federico II.”

Exclusion criteria were the following: chronic inflammatory diseases that could interfere with the systemic or local inflammatory profile, history of AF. Before cardiac surgery, all patients underwent clinical and echocardiographic assessment. Clinical and demographic data were recorded, including cardiovascular risk factors and drug therapies. According to standard techniques, echocardiograms were performed by a Vivid E9 (GE Healthcare) machine. EAT was visualized in a parasternal long-axis view between the free wall of the right ventricle and the anterior surface of the ascending aorta. Once visualized the EAT deposit, the maximum EAT thickness was measured at end-systole, as previously described (Parisi et al., 2020a). The average value from three cardiac cycles was used for the statistical analysis.

Before cardiac surgery, we collected blood samples for serum collection. Intraoperative EAT biopsies (average 0.1–0.5 g) and right atrial appendages were carried out before the initiation of cardiopulmonary bypass. EAT biopsies were taken between the free wall of the right ventricle and the anterior surface of the ascending aorta, just after the opening of the pericardial sac. Each tissue sample was stored at −80°C until analysis. All patients were postoperatively monitored by ECG telemetry and data on POAF occurrence were recorded during the hospital stay.

Informed consent was obtained from every subject before the surgical procedure. Protocols were approved by the ethical committee of the University of Naples (prot. no. 301/19). All procedures performed in the study were in accordance with the ethical standards of the institutional or national research committee and with the 1964 Helsinki declaration and its later amendments or comparable ethical standards and conformed to the Declaration of Helsinki on human research.

### Conditioned Media Preparation and Cytokines Assessment

According to tissue weight, serum-free Dulbecco modified Eagle medium (DMEM)-F12 (1:1) containing 0.25% BSA (1 ml medium/0.1 g tissue) was added to the well and incubated at 37°C in a CO2 incubator. After 24 h, the medium was collected, centrifuged at 14,000 × g to remove debris, and then stored as aliquots at –80°C until further use. Serum samples and conditioned media were screened for the concentration of interleukin (IL)-1ra, IL-1b, IL-2, IL-4, IL-5, IL-6, IL-7, IL-8, IL-9, IL-10, IL-12 (p70), IL-13, IL-15, IL-17A, basic fibroblast growth factor (FGF), eotaxin, granulocyte-colony stimulating factor (G-CSF), granulocyte macrophage-colony stimulating factor (GM-CSF), interferon-γ (IFN-γ), interferon-γ inducible protein 10 (IP-10), monocyte chemoattractant protein-1 (MCP-1), macrophage inflammatory protein-1 (MIP-1) α, MIP-1β, C–C motif chemokine ligand 5 (CCL5)/RANTES, TNF-α, platelet-derived growth factor (PDGF-BB) and vascular endothelial growth factor (VEGF) using the Bio-Plex Pro Human Cytokine Grp I Panel 27-Plex kit (cat. no. M500KCAF0Y) according to the supplier’s instructions. The magnetic bead-based assay was performed on a Bio-Plex 200 analyzer with Bio-Rad Bio-Plex Manager (Bio-Rad, Hercules, CA, United States).

### Atrial Fibrosis Assessment at Histology

Fresh tissue from atrial biopsies was formalin-fixed and paraffin-embedded. For each case 4 µm-thick sections were cut and stained with hematoxylin/eosin and observed on a light microscope to evaluate fibrosis, graded as 0, 1+, 2+ and 3+, respectively when absent or observed in <10%, 10–50% and in >50% of the sample. The volume fraction of collagen (CVF) was analyzed by staining with hematoxylin/eosin. Four separate views were selected (magnification = original ×400) and the CVF was calculated using the following formula: CVF = collagen area/total visual area × 100%, to assess the degree of cardiac fibrosis. Each sample was independently evaluated by two different pathologists.

### Cell Culture and Treatments

Human fibroblasts were isolated from the right atrial auricle by mechanical and enzymatic digestion (collagenase type II) of the tissue and cultured in Advanced Dulbecco’s modified eagle’s medium (Ad DMEM; Life Technologies) with 10% fetal bovine serum (FBS; Microtech, IT), 1% penicillin (Life Technologies), 1% streptomycin (Life Technologies), and 1% L-glutamine (Life Technologies). Primary cells were immortalized by transduction with commercial lentiviral particles carrying out the encoding sequences for SV40 large T antigen and human telomerase reverse transcriptase (hTERT) enzyme, under cytomegalovirus promoter (GeneCopoeia). Interleukin (IL) treatments were performed using human recombinant IL-1β (R&D Systems, Inc., Minneapolis, MN, United States) at 0.1, 1, and 10 ng/ml and IL-18 (R&D Systems, Inc., Minneapolis, MN, United States) at 1, 10, and 100 ng/ml. FBS at 10% was used as a positive control for migration assay.

### MTT Assay

To evaluate the proliferation rate, 3000 cells per well were plated in a 96 well plate and followed by Incucyte (Essen BioScience) every 8 h for 5 days. Cells were treated with recombinant ILs (as previously reported) every other day. At the end of the survey, cell viability was assessed by 3-(4, 5-dimethyl thiazolyl-2)-2,5-diphenyltetrazolium bromide (MTT; Sigma-Aldrich). Briefly, MTT solution (0.5 mg/ml) was added into each well and incubated at 37°C, 5% CO2 for 4 h. The supernatant was discarded and dimethylsulfoxide (DMSO; Sigma-Aldrich) was added into each well. The corresponding absorbance value was observed using the microplate reader (TECAN pro infinite M200) at a dual-wavelength of 590 and 620 nm (reference). Cell viability was expressed as a percentage with respect to the mean of untreated, referred to as 100%.

### Migration Assay

To evaluate the migration, 5,000 cells per well were plated in a 96 well plate and starved overnight in Ad DMEM without FBS. The day after, a scratch was performed using wound maker 96 (Essen BioScience), treatments with ILs were added and wound healing was evaluated by Incucyte (Essen BioScience) every 2 h for 1 day. Relative wound density was expressed as a percentage referred to the same well at time 0.

### RNA Isolation and Expression Analysis

Total RNA was isolated from EAT biopsies and cell culture, using TRIzol solution (Life Technologies, CA, United States), quantified (NanoDrop spectrophotometer, Life Technologies, Carlsbad, CA, United States), and reverse-transcribed using SuperScript III Reverse Transcriptase according to the manufacturer’s instructions. qPCR was performed by iTaq Universal SYBR Green Supermix (Biorad). Absolute quantification of gene expression (Arbitrary Units—AU) was measured by using the 2^−ΔCt^ method (EAT biopsies). Relative quantification (AU) of gene expression was measured by using 2^−ΔΔCt^ method (cell culture). Expression levels of *IL-1β* (*IL-1β* primer pairs: F-5′-ACTGAAAGCTCTCCACCTCC-3’; R-5′-CATCTTTCAACACGCAGGAC-3′), Collagen 1A1 (*COL1A1* primer pairs: F-5′-GGACACAGAGGTTTCAGTGG-3’; R-5′-CCAGTAGCACCATCATTTCC-3′), Collagen 3A1 (*COL3A1* primer pairs: F-5′-CTACTTCTCGCTCTGCTTCATC-3’; R-5′-TTGGCATGGTTCTGGCTT-3′), Transforming Growth Factor β1 (*TGFβ1* primer pairs: F-5′-GTTCAGGTACCGCTTCTCG-3’; R-5′-CCGACTACTACGCCAAGGA-3′) were normalized for the reference sample using Peptidylprolyl Isomerase A (*PPIA*) as housekeeping gene (*PPIA* primer pairs: F-5′-TACGGGTCCTGGCATCTTGT-3’; R-5′-GGTGATCTTCTTGCTGGTC-3′).

### Statistical Analysis

Statistical analyses were performed with R statistical platform and with GraphPad Prism 8.0 software (GraphPad Software Inc., La Jolla, CA). D’Agostino-Pearson normality test was used to evaluate whether the continuous data were normally distributed, and according to the results, a Welch’s two-tailed *t*-test for independent samples (for normally distributed data) or a Mann-Whitney *U* test (for not normally distributed data) was used. Categorical values were described by the number of occurrences and percentages and compared by the chi-square test. One-way analysis of variance (ANOVA) followed by Tukey’s multi comparison test was used for the *in vitro* data.

Receiver-operating characteristics (ROC) curves were used to evaluate IL-1β′s ability to classify POAF and no-POAF patients.

## Results

### Patient Clinical Characteristics and Atrial Fibrosis

Forty CAD patients were selected for the study (mean age 61 years, male gender 95%). No differences in surgical procedures were noted in the two groups, and in all patients extracorporeal circulation was performed. Table 1 illustrates the demographic, clinical, and echocardiographic characteristics of overall patients. Sixteen patients out of 40 (40%) developed AF during the 7 days following the cardiac surgery (POAF group), while 60% exhibited constant sinus rhythm in the postoperative period (no-POAF). The two groups were homogeneous concerning age, clinical and echocardiographic parameters, including left atrial volume and EAT thickness (Table 1). In a subgroup of 20 patients, we assessed atrial fibrosis at histology. The 60% of patients has grade 0 (no fibrosis), the 5% had grade 1 (<10% fibrosis), the 25% had grade 2 (10–50% fibrosis) and 10% had grade 3 (>50%) (Supplementary Figure S1). Overall atrial fibrosis was present in eight patients. Of note, no association was observed between POAF and the presence of atrial fibrosis (*p* = 0.582, data not shown).

**TABLE 1:** Patient clinical characteristics in No-POAF and POAF groups.

	No-POAF (*n* = 24)	POAF (*n* = 16)	*p*-value (Test)
Sex (M) (%)	22 (91.67%)	16 (100%)	0.236^a^
Age (years)	61 ± 10.5	62.1 ± 9.51	0.717^b^
BMI (Kg/m^2^)	29.4 (27.7; 31.5)	29.4 (27.4; 30.4)	0.445^b^
Diabetes (%)	4 (20%)	6 (37.5%)	0.136^a^
LVEF (%)	55 (51; 60)	55 (48; 59.5)	0.445^c^
E/A	0.72 (0.68; 0.83)	0.805 (0.61; 0.975)	0.712^c^
E/E′	8.8 (6.52; 10)	9.76 (7.22; 11.4)	0.595^b^
EAT thickness (mm)	14.5 (12.3; 16.8)	14.5 (12; 17)	0.904^b^
Left Atrial Volume (ml/m^2^)	32.5 (28.5; 45.8)	37 (31.3; 43.8)	0.439^c^
Creatinine (mg/dl)	0.89 (0.80; 1.04)	0.96 (0.78; 1.27)	0.677^c^
GFR (ml/min)	87 (75.25; 99.75)	80 (59; 93)	0.250^b^
Hypertension (%)	21 (87.5%)	16 (100%)	0.141^a^
CRP (mg/L)	1.75 (0.825; 4.85)	1.2 (0.75; 4.1)	0.617^c^
Blood Glucose (mg/dl)	97 (89.25; 115)	107.5 (96; 168.8)	0.068^b^
Sodium (mmol/L)	141 (140; 142)	140 (138; 141)	0.066^b^
Potassium (mmol/L)	4.5 (4.125; 4.7)	4.3 (4.025; 4.7)	0.745^b^
Calcium (mg/dl)	8.95 (8.8; 9.375)	9.2 (8.925; 9.4)	0.367^c^
INR	1.06 (0.96; 1.10)	1.06 (1.02; 1.07)	0.178^b^
aPTT (sec)	29.95 (28.18; 32.65)	30 (28.18; 33.18)	0.736^b^
Serum IL-1β (pg/ml)	3.14 (2.83; 3.53)	3.24 (2.86; 3.34)	0.767^c^
Serum IL-1ra (pg/ml)	1072 (1025; 1176)	1132 (1025; 1191)	0.527^c^
EAT IL-1β (pg/ml)	0.86 (0.76; 0.985)	1.04 (0.873; 1.43)	**0.009** ^c^
EAT IL-1ra (pg/ml)	452 (326; 623)	654 (361; 781)	0.121^b^

Results are expressed as median and range (25% percentile; 75% percentile) or as the number of cases (%). Age is expressed as media ±SD. BMI, body mass index; LVEF, left ventricular ejection fraction; EAT, epicardial adipose tissue; IL, interleukine; GFR, glomerular filtration rate; CRP, C-reactive protein; INR, international normalized ratio; aPTT, activated partial thromboplastin time.

Statistically significative values (p < 0.05) are reported in bold.

a Chi Squared test.

b Unpaired t-test.

c Mann-Whitney U test.

### Circulating and EAT-Derived Interleukins

**FIGURE 1:**
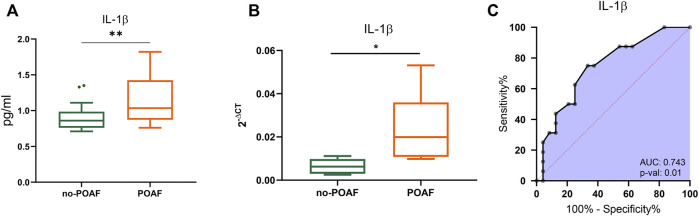
IL-1β in POAF subgroups. **(A)** Boxplots denote IL-1β concentration distributions in conditioned media from EAT biopsies of subjects with the no-POAF outcome (“no-POAF”—green) and with POAF outcome (“POAF”—orange); IL-1β concentration is expressed as pg/ml. Box plots denote median and 25th–75th percentiles (boxes) and tukey whiskers. **(B)** Boxplots denote IL-1β absolute expression in EAT biopsies for 6 subjects with no POAF outcome (“no-POAF”—green) and 6 with POAF outcome (“POAF”—orange). Box plots denote median and 25th–75th percentiles (boxes) and min-to-max whiskers. **(C)** AUC of ROC analysis indicates the performance of IL-1β. *p*-value refers to the significant difference from the AUC basal level of 0.5 (red dotted line). *p < 0.05; **p < 0.01.

No significant difference was observed in serum levels of IL-1β, IL-1ra and other cytokines, chemokines, and growth factors between POAF and no-POAF groups [IL-1β: 3.24 pg/ml (2.86; 3.34) *vs.* 3.14 pg/ml (2.83; 3.53); IL-1ra: 1132 pg/ml (1025; 1191) *vs.* 1072 pg/ml (1025; 1176), Table 1 and Supplementary Table S1]. To gain insight into a possible involvement of local inflammation in POAF occurrence, we obtained conditioned media from EAT biopsies and analyzed a panel of inflammatory mediators. IL-1β local levels were significantly higher in the POAF group compared to the no-POAF group (*p* = 0.009) (Figure 1A; Table 1). In parallel, no significant differences were observed in EAT-derived IL-1ra between the two groups (Table 1). Consistently, EAT-derived IL-2, IL-5, IL-6, IL-8, and IP-10 levels were significantly higher in the POAF group (Supplementary Table S1). To note, according to secretion, also *IL-1β* mRNA expression was significantly higher in EAT biopsies from POAF patients compared to no-POAF (*p* = 0.0308) (Figure 1B).

ROC analyses revealed that IL-1β provides valuable discrimination between the two groups (AUC 0.743, 95% CI: 0.588–0.899, *p* = 0.01) (Figure 1C).

### Interleukins’ Effects on Human Cardiac Fibroblasts

**FIGURE 2:**
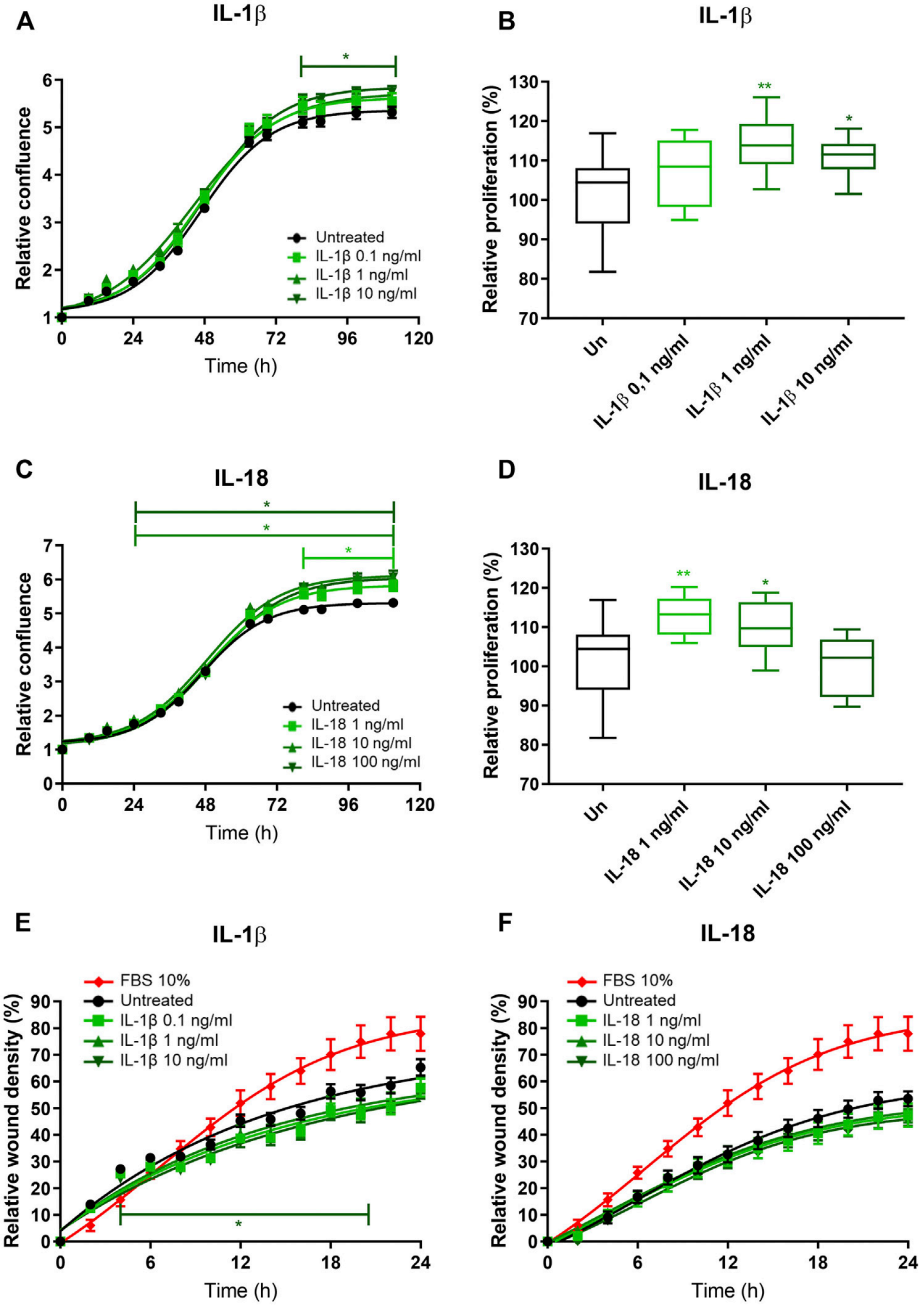
IL-1β and IL-18 effects on cardiac fibroblast proliferation and migration. **(A–C)** Relative confluence normalized on T0 and **(B–D)** relative proliferation with respect to the untreated (setted to 100%) and **(E,F)** relative wound healing density normalized on T0 of human immortalized fibroblasts exposed to different concentrations of **(A,B–E)** IL-1β and **(C,D–F)** IL-18 (*n* = 8). Data are expressed by mean ± SEM. *p < 0.05; **p < 0.01 vs. untreated.

To investigate the potential role of EAT-secreted IL-1β on atrial remodeling and fibrosis, we *in vitro* tested the effects of IL-1β on human immortalized fibroblasts obtained from the right atrium of patients undergoing cardiac surgery. The relative confluence of fibroblasts exposed to increasing concentrations of recombinant IL-1β was higher compared to the untreated, at the highest tested concentration (10 ng/ml) starting from the third day (*p* < 0.05; Figure 2A). MTT assay revealed that the proliferation rate of fibroblasts exposed to IL-1β was higher than the control at the concentration of 1 ng/ml (114.0% ± 7.1% vs. 101.6% ± 9.8%; *p* = 0.003) and 10 ng/ml (111.0% ± 5.2%; *p* = 0.03), while the lower tested concentration (0.1 ng/ml) had no effect (107.7% ± 8.7%; *p* = 0.13; ANOVA *p* = 0.004; Figure 2B). As IL-1β and IL-18 are members of the same structural family and are involved in the NLRP3 inflammasome, we also tested the *in vitro* effects of IL-18 on fibroblasts. IL-18 induced an increased relative confluence than control, even at the lowest tested concentration (1 ng/ml; Figure 2C). Indeed, the proliferation rate of cells exposed to IL-18 was higher than control (1 ng/ml 112.9% ± 5.0% vs. 10 ng/ml 109.8% ± 6.8% vs. 100 ng/ml 99.9% ± 7.5% *vs.* control 101.6% ± 9.8%; ANOVA *p* = 0.003; Figure 2D). To better characterize the effects of these ILs on human fibroblasts, we analyzed the migration rate. IL-1β reduced the invasion capacity of fibroblasts at the highest tested concentration (10 ng/ml; *p* = 0.05; Figure 2A), while IL-18 did not have any effects on wound healing capacity at any analyzed concentration (Figures 2E,F).

**FIGURE 3:**
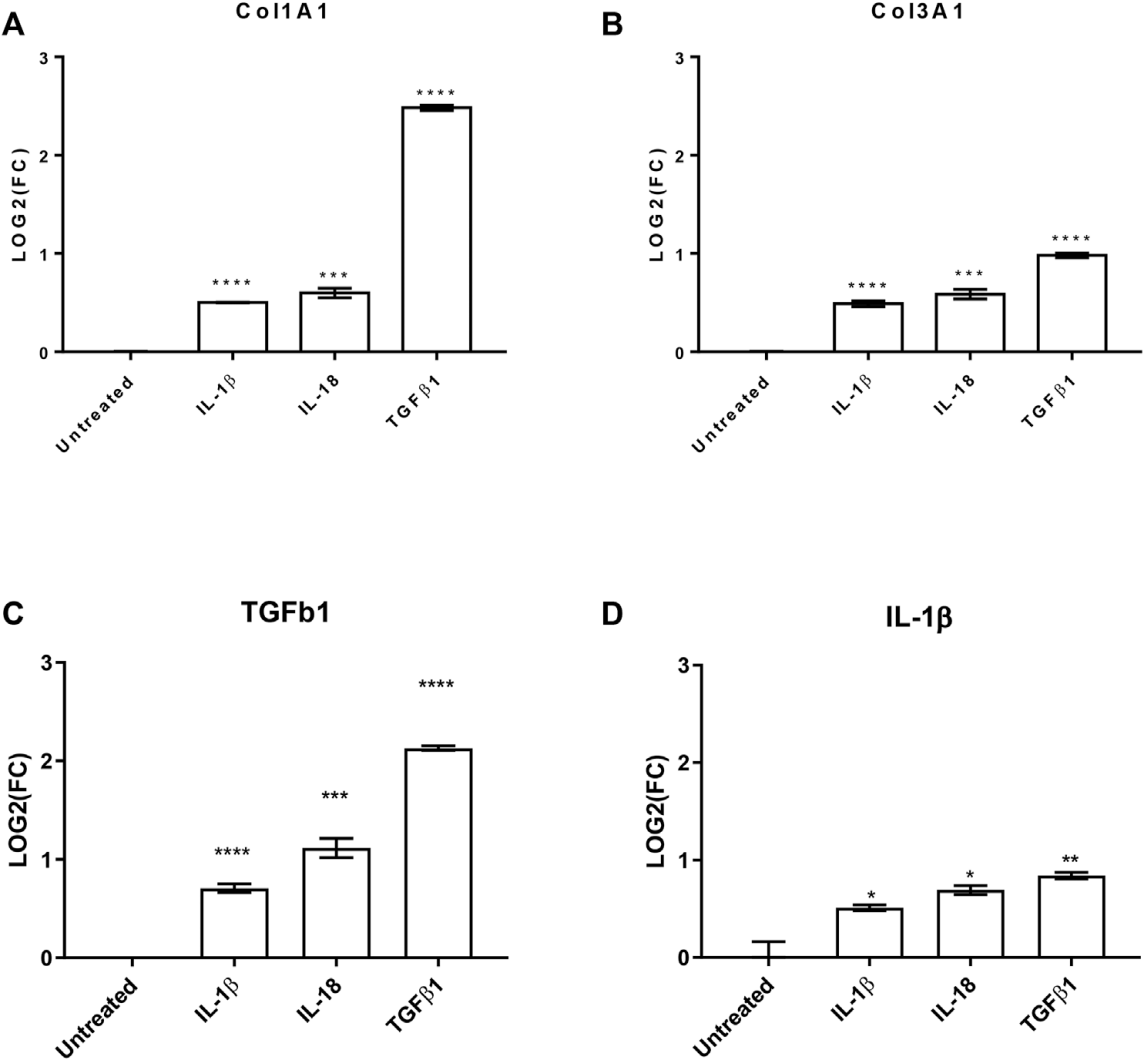
IL-1β and IL-18 effects on pro-fibrotic and pro-inflammatory genes. Bar graphs reporting the relative gene expression of **(A)** collagen 1A1 (Col1A1), **(B)** collagen 3A1 (Col3A1), **(C)** transforming growth factor β1 (TGFβ1), and **(D)** interleukin 1β (IL-1β) of human cardiac fibroblasts under IL-1β, IL-18, and TGFβ1 (as positive control) treatments (*n* = 3). Data are expressed by mean ± SEM. *p < 0.05; **p < 0.01; ***p < 0.001; ****p < 0.0001 vs. untreated.

To further evaluate the effects of these interleukins on pro-fibrotic and pro-inflammatory effects on cardiac fibroblast, we investigated by qPCR the production of collagen 1A1 (*COL1A1*), *COL3A1*, and transforming growth factor β 1 (*TGFβ1*) as well as the production of *IL-1β*. All the analyzed transcripts were significantly upregulated by both the used interleukins (Figure 3). Interestingly, the treatments increased *TGFβ1* production, which is the most powerful pro-fibrotic inducer, and at the same time potentiate the pro-inflammatory effects.

In conclusion, IL-1β and IL-18 improved the proliferation rate of human cardiac fibroblasts potentiating the fibrosis machinery with a rebound on the inflammation itself.

## Discussion

The present study explored the potential role of EAT-secreted IL-1β in atrial remodeling and POAF occurrence in patients undergoing CABG, without a history of AF. We observed: 1) POAF onset is not associated with clinical nor echocardiographic parameters; 2) In patients with and without POAF there are no differences in EAT thickness, atrial dilation, and fibrosis; 3) EAT-IL1β secreted levels, but not circulating IL-1β levels are associated with POAF occurrence; 4) NLRP3 inflammasome cytokines, IL1β and IL-18 could promote atrial fibrosis.

In patients undergoing cardiac surgery, POAF is a frequent complication and occurs approximately in 20–40% of cases (Dobrev et al., 2019). It is a clinically relevant event, being associated with hemodynamic instability, increased risk of stroke, lengthened hospital and intensive care unit stays, and greater hospital costs (Dobrev et al., 2019). POAF is also a predictor of permanent AF. However, the mechanisms underlying AF and POAF are probably different as POAF is mainly driven by periprocedural triggers. Cardiac surgery itself contributes to the pathophysiology of AF, both in the initiation and maintenance of arrhythmia (Hu et al., 2015). In particular, POAF occurs in presence of factors inducing atrial arrhythmogenic remodeling before surgery and is promoted by peri-operative triggers. In some studies, POAF is associated with preoperative structural alterations including interstitial fibrosis (Maesen et al., 2012). However, in the present study neither left atrial volume, evaluated at echocardiography nor atrial fibrosis, evaluated at histology, were associated with POAF occurrence. Similarly, in our population, we didn’t observe differences in demographic, clinical, and echocardiographic parameters among patients with and without POAF. Thus, here, we can exclude a possible association of POAF with clear predisposing factors.

Inflammation is considered a major trigger for POAF, even if the association between levels of inflammatory cytokines and the occurrence of POAF is not well defined. Recently, it has been reported a strong association between EAT-derived activin A expression and atrial remodeling (Venteclef et al., 2015; Wang et al., 2019). Of note, it has been shown that there is a direct link between activin A and IL-1β in the contest of inflammation and fibrosis. Moreover, this link is bidirectional since the former is able to stimulate the latter expression and *vice versa* (de Kretser et al., 2012). In this context, our study, focusing on IL-1β, is in accordance with the literature and adds a brick to the wall of knowledge regarding inflammatory cytokines involvement and POAF onset. Of note, in our population, there was no significant differences in circulating levels of cytokines, chemokines and growth factors evaluated. In particular, no variation in circulating levels of IL-1β and IL-1ra between POAF and no-POAF patients, raising the hypothesis of a specific role of EAT and local inflammation in POAF.

EAT is the visceral fat depot of the heart, and it is located between the myocardium and the visceral layer of the pericardium. In physiological conditions, EAT has several protective functions on the heart; however, it is now well established that it can also have undesirable effects on cardiac tissue. In absence of fascial boundaries, EAT directly influences the myocardial electrical and structural remodeling and it is associated with cardiovascular diseases (Mazurek et al., 2003; Chaldakov et al., 2014; Parisi et al., 2015; Ghenev et al., 2016). The release of proinflammatory cytokines by EAT is initiated by the innate inflammatory response through Toll-like receptors (TLRs) located in the macrophages, B cells, dendritic cells, and adipocyte membranes. The receptors recognize antigens such as lipopolysaccharide (LPS) and saturated fatty acids, promoting nuclear factor kappa-beta (NF-*κβ*) translocation into the nucleus of epicardial adipocytes, with transcription of inflammatory mediators such as IL-1, IL-8, IL-6, and TNF-*α*, linking innate immunity and chronic inflammatory responses. We previously described that EAT is a source of IL-1β in CAD patients, and the imbalance between levels of IL‐1β and its receptor antagonist (IL‐1ra) drives cardiovascular events (Parisi et al., 2020b).

Our data indicate that EAT inflammatory profile is increased with POAF occurrence. In particular, EAT-mediated IL-1β secretion and mRNA expression are higher in POAF patients and IL-1ra secretion doesn’t differ in the two groups, indicating an increased pro-inflammatory effect of IL-1β in POAF patients. To note, all biopsies were collected at the surgical time, before extracorporeal circulation to avoid possible inflammation of tissues mediated by surgical manipulation. Along with the evidence that local inflammatory status could give rise to abnormal atrial conduction (Mariscalco and Engström, 2008), we have hypothesized that POAF patients may have a pre-existing local inflammatory state characterized by enhanced IL-1β, an essential component of the NLRP3-inflammasome signaling. The NLRP3- inflammasome is a deeply rooted signaling pathway responsible for IL-1β and IL-18 releases from innate immune cells (Heijman et al., 2020).

Patients with POAF are considered to be at higher risk of permanent AF (Dobrev et al., 2019), thus it is plausible that factors that trigger POAF could promote atrial remodeling and fibrosis over time. To verify this hypothesis, we mimicked post-operative inflammation by *in vitro* stimuli of NLRP3-related cytokines, IL-1β and IL-18, on human fibroblasts isolated from atrial myocardium. We observed that both IL-1β and IL-18 promote fibroblasts proliferation. In patients with AF undergoing left atrial appendage excision, EAT-derived IL-1β was associated with the total atrial collagen content (Abe et al., 2018). However, no evidence is available on POAF patients. Animal studies conducted on mice suggested that IL-18 stimulates fibroblast migration and proliferation (Fix et al., 2011). Moreover, recent evidence supports the hypothesis that pre-existing activation of the atrial cardiomyocyte NLRP3-inflammasome contributes to the POAF-predisposing substrate (Heijman et al., 2020). Here, we provide the first evidence that the paracrine release of IL-1β from EAT could promote POAF, even in absence of pre-operative predisposing factors. Furthermore, we observed that inflammatory stimuli on atrial fibroblasts promote the production of collagen and TGFβ1, the most powerful pro-fibrotic inducer, and at the same time potentiate the pro-inflammatory effects. All the way, our observations give convincing proof that IL-1β could be an important mediator of the pro-fibrotic effect of EAT on atrial remodeling as already described for Activin A (Wang et al., 2019) and Galectin-3 (Rhodes et al., 2013; Fashanu et al., 2017).

This intensive crosstalk between EAT-derived molecules and the surrounding myocardium provides novel insights into the potential establishment of a pro-fibrotic milieu and supports promoting changes in the atrial myocardium. These results pay the way for new studies exploring the potential benefits of anti-inflammatory drugs in the perioperative period and suggest that EAT could mediate the antiarrhythmic effects observed by using anti-inflammatory therapies in animal studies (Wu et al., 2020).

### Study Limitations

The present study evaluated the predictive value of pre-operative circulating inflammatory markers in POAF occurrence. We didn’t explore the predictive value of post-operative inflammatory markers. However, since cardiac intervention is an important inflammatory trigger, a dedicated study should be designed to appropriately identify the impact of post-operative inflammatory markers in POAF occurrence.

We *in vitro* tested the effects of single cytokines on fibroblast proliferation and collagen production. Further studies are required to test the effects of the entire EAT-secretome on cardiac cells and the potential benefits of IL-1β inhibition.

Genetic variation and heritability take part in AF onset (Ellinor et al., 2012; Kolek et al., 2015). Genome-wide association studies have identified several common variant *loci* associated with AF and functionally implicated in cardiac development, electrophysiology, and cardiomyocyte behavior (Roselli et al., 2020). Surely, adding information on AF genetic variability and/or on common risk Single Nucleotide Polymorphisms (SNPs) would improve insight in our population.

## Data Availability

The original contributions presented in the study are included in the article/Supplementary Material, further inquiries can be directed to the corresponding authors.
