# Supplementary material for: Epicardial Adipose Tissue-Derived IL-1β Triggers Postoperative Atrial Fibrillation
Source: Front Cell Dev Biol. 2022 May 5;10:893729. doi: 10.3389/fcell.2022.893729 (PMC9198900; doi:10.3389/fcell.2022.893729)
Supplement: Supplementary file 1 [file Table1.DOCX]

| SERUM | | | | EAT-SECRETOME | | | |
| --- | --- | --- | --- | --- | --- | --- | --- |
|  | **No POAF (n=24)** | **POAF (n=16)** | **p-val  (test)** |  | **No POAF (n=24)** | **POAF (n=16)** | **p-val  (test)** |
| IL-1β | 3.14 [2.83; 3.53] | 3.24 [2.86; 3.34] | 0.767^b^ ns | **IL-1β** | 0.86 [0.76; 0.985] | 1.04 [0.873; 1.43] | **0.009^b^ **** |
| IL-1ra | 1072 [1025; 1176] | 1132 [1025; 1191] | 0.527^b^ ns | **IL-1ra** | 452 [326; 623] | 654 [361; 781] | 0.121^a^ ns |
| IL-2 | 13.1 [11.7; 15.2] | 13.6 [12.7; 14.7] | 0.569^b^ ns | **IL-2** | 4.28 [3.75; 4.78] | 4.69 [4.3; 5.45] | **0.048^b^ *** |
| IL-4 | 3.61 [3.41; 4.21] | 3.81 [3.41; 4.17] | 0.706^b^ ns | **IL-4** | 0.71 [0.57; 0.91] | 0.89 [0.61; 1.47] | 0.341^b^ ns |
| IL-5 | 182 [146; 254] | 165 [131; 234] | 0.439^b^ ns | **IL-5** | 98.9 [83.2; 109] | 116 [101; 130] | **0.015^b^ *** |
| IL-6 | 12.7 [9.39; 20.4] | 14 [9.13; 16.8] | 0.302^a^ ns | **IL-6** | 32.8 [17.4; 69.7] | 67.2 [20.9; 248] | **0.044^a^ *** |
| IL-7 | 15 [12.9; 20] | 16.4 [15; 20.3] | 0.293^b^ ns | **IL-7** | 3.06 [2.31; 3.06] | 3.24 [3.06; 3.76] | 0.106^a^ ns |
| IL-8 | 27.1 [22.2; 41.5] | 26.7 [22.4; 45.2] | 0.897^b^ ns | **IL-8** | 54.5 [20.3; 119] | 138 [107; 299] | **0.025^b^ *** |
| IL-9 | 568 [404; 675] | 628 [437; 679] | 0.583^a^ ns | **IL-9** | 14.7 [10.4; 19.2] | 19.4 [12.5; 24.3] | 0.108^b^ ns |
| IL-10 | 19.3 [15.9; 24.5] | 17.6 [14.9; 21.9] | 0.095^a^ ns | **IL-10** | 6.11 [5.72; 6.54] | 6.33 [5.53; 8.68] | 0.414^b^ ns |
| IL-12 | 14.4 [12.8; 16.7] | 14.1 [13.2; 15.2] | 0.691^a^ ns | **IL-12** | 3.88 [3.6; 4.17] | 3.95 [3.6; 4.38] | 0.722^a^ ns |
| IL-13 | 2.64 [2.64; 2.97] | 2.64 [2.64; 3.21] | 0.781^b^ ns | **IL-13** | 0.74 [0.74; 0.82] | 0.82 [0.74; 0.88] | 0.435^b^ ns |
| IL-15 | 883 [755; 989] | 819 [699; 967] | 0.701^a^ ns | **IL-15** | 294 [247; 313] | 314 [282; 351] | 0.102^a^ ns |
| IL-17 | 20.5 [17.7; 23.3] | 21.2 [18.8; 23.1] | 0.331^b^ ns | **IL-17** | 3.94 [3.22; 4.59] | 4.3 [3.7; 6.19] | 0.145^b^  ns |
| Eotaxin | 58.4 [42.2; 65.8] | 55.3 [41.3; 77.4] | 0.929^b^ ns | **Eotaxin** | 0.85 [0.7; 0.983] | 1.12 [0.785; 1.42] | 0.171^a^ ns |
| FGF-basic | 66.3 [59.2; 70.6] | 65.6 [63.7; 70.6] | 0.832^b^ ns | **FGF-basic** | 357 [227; 527] | 336 [169; 509] | >0.999^b^ ns |
| G-CSF | 227 [146; 334] | 208 [175; 315] | 0.769^b^ ns | **G-CSF** | 62.9 [32.3; 113] | 158 [35; 352] | 0.128^b^ ns |
| GM-CSF | 4.71 [3.97; 4.98] | 4.44 [4.17; 4.95] | 0.864^b^ ns | **GM-CSF** | 1.41 [1.28; 1.62] | 1.57 [1.26; 1.77] | 0.555^a^ ns |
| IFN-γ | 16.1 [15.3; 17.7] | 16.9 [15.4; 17.7] | 0.539^b^ ns | **IFN-γ** | 4.43 [3.74; 5.56] | 5.26 [4.23; 7.05] | 0.069^b^ ns |
| IP-10 | 353 [289; 802] | 524 [364; 789] | 0.192^b^ ns | **IP-10** | 13.8 [13; 14.2] | 14.3 [13.8; 19.8] | **0.007^b^ **** |
| MCP-1 | 54.5 [43.6; 77.8] | 53.1 [45.4; 78.4] | 0.398^a^ ns | **MCP-1** | 104 [39; 158] | 123 [61.1; 277] | 0.539^b^ ns |
| MIP-1α | 5.61 [3.91; 11] | 5.85 [4.92; 9.53] | 0.638^b^ ns | **MIP-1α** | 1.74 [0.763; 4.15] | 2.73 [1.05; 9.72] | 0.385^b^ ns |
| MIP-1β | 297 [229; 334] | 298 [247; 327] | 0.989^b^ ns | **MIP-1β** | 10.3 [6.34; 20.8] | 16.1 [6.88; 35.5] | 0.185^b^ ns |
| PDGF | 520 [297; 881] | 686 [220; 1128] | 0.613^b^ ns | **PDGF** | 16.7 [15.5; 30.8] | 21.7 [19; 29.3] | 0.209^b^ ns |
| RANTES | 10044 [5372; 12315] | 10878 [5507; 13485] | 0.614^a^ ns | **RANTES** | 163 [72.8; 239] | 127 [37.9; 304] | 0.557^b^ ns |
| TNF-α | 44.3 [37.5; 58.5] | 51.1 [46.4; 60.2] | 0.154^b^ ns | **TNF-α** | 7.64 [3.94; 9.28] | 7.8 [5.4; 13.2] | 0.127^b^ ns |
| VEGF | 358 [307; 509] | 358 [276; 453] | 0.481^b^ ns | **VEGF** | 148 [122; 164] | 165  [136; 185] | 0.053^b^ ns |
| a: unpaired t-test; b: Mann-Whitney U test | | | | | | | |

Values are indicated as pg/ml. Results are expressed as median and range [25% percentile; 75% percentile]; IL: Interleukine.
