# Supplementary figures and images for: Epicardial Adipose Tissue-Derived IL-1β Triggers Postoperative Atrial Fibrillation
Source: Front Cell Dev Biol. 2022 May 5;10:893729. doi: 10.3389/fcell.2022.893729 (PMC9198900; doi:10.3389/fcell.2022.893729)

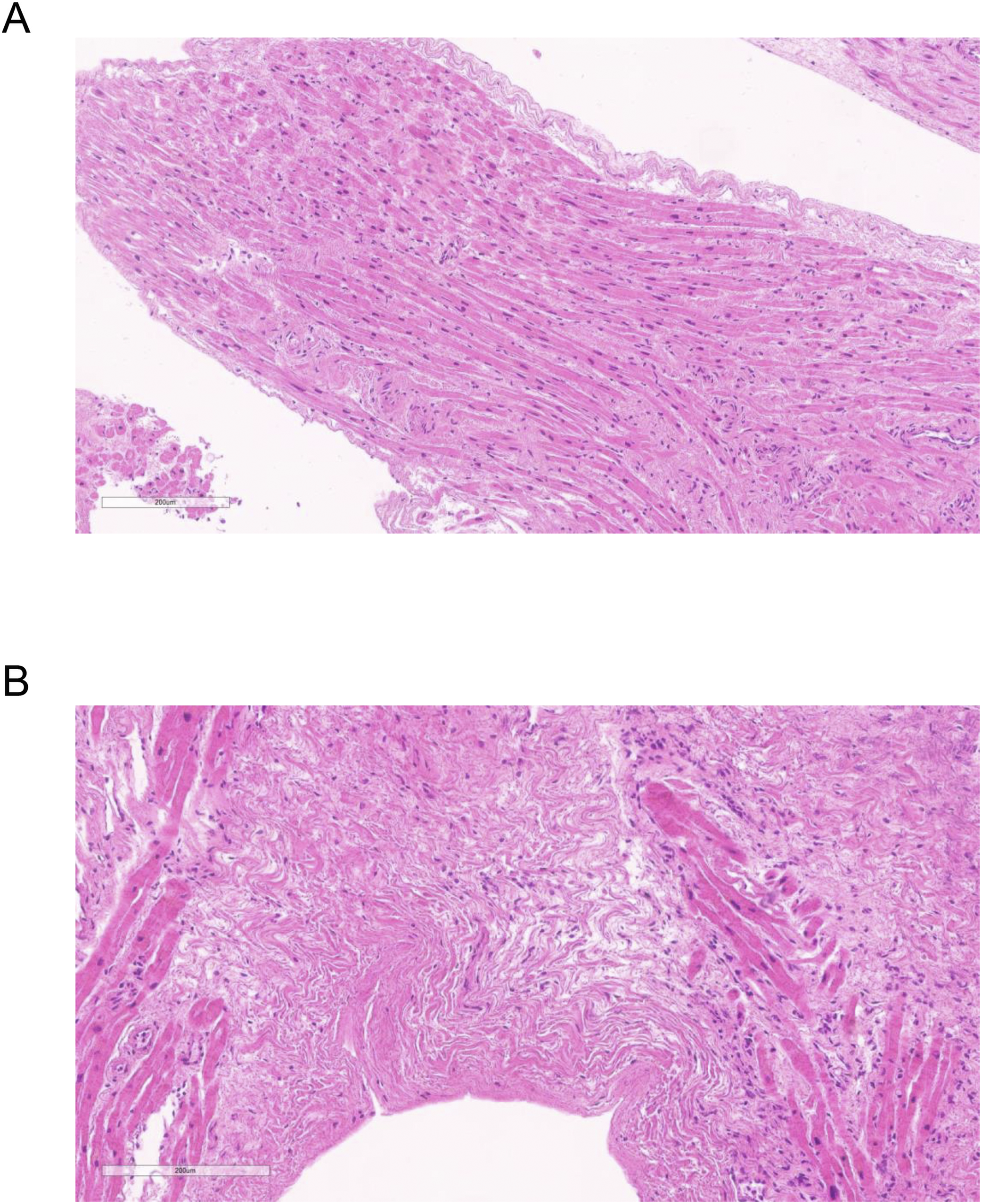

Supplement: Supplementary file 2 [file Image1.tif]
